# Functional integration of natural killer cells in a microfluidically perfused liver on-a-chip model

**DOI:** 10.1186/s13104-023-06575-w

**Published:** 2023-10-21

**Authors:** René Fahrner, Marko Gröger, Utz Settmacher, Alexander S. Mosig

**Affiliations:** 1 Department of General, Visceral and Vascular Surgery, Jena University Hospital, 07747 Jena, Germany; 2 Department of Vascular Surgery, University Hospital Bern, University of Bern, 3010 Bern, Switzerland; 3 Integrated Research and Treatment Center, Center for Sepsis Control and Care (CSCC), Jena University Hospital, 07747 Jena, Germany

**Keywords:** Natural killer cells, NKp46, Liver, Microfluidic, Liver-on-a-chip, Cytokines

## Abstract

**Objective:**

The liver acts as an innate immunity-dominant organ and natural killer (NK) cells, are the main lymphocyte population in the human liver. NK cells are in close interaction with other immune cells, acting as the first line of defense against pathogens, infections, and injury.

A previously developed, three-dimensional, perfused liver-on-a-chip comprised of human cells was used to integrate NK cells, representing pivotal immune cells during liver injury and regeneration. The objective of this study was to integrate functional NK cells in an in vitro model of the human liver and assess utilization of the model for NK cell-dependent studies of liver inflammation.

**Results:**

NK cells from human blood and liver specimen were isolated by Percoll separation with subsequent magnetic cell separation (MACS), yielding highly purified blood and liver derived NK cells. After stimulation with toll-like-receptor (TLR) agonists (lipopolysaccharides, Pam3CSK4), isolated NK cells showed increased interferon (IFN)-gamma secretion. To study the role of NK cells in a complex hepatic environment, these cells were integrated in the vascular compartment of a microfluidically supported liver-on-a-chip model in close interaction with endothelial and resident macrophages. Successful, functional integration of NK cells was verified by immunofluorescence staining (NKp46), flow cytometry analysis and TLR agonist-dependent secretion of interleukin (IL)-6 and tumor necrosis factor (TNF)-alpha. Lastly, we observed that inflammatory activation of NK cells in the liver-on-a-chip led to a loss of vascular barrier integrity. Overall, our data shows the first successful, functional integration of NK cells in a liver-on-a-chip model that can be utilized to investigate NK cell-dependent effects on liver inflammation in vitro.

**Supplementary Information:**

The online version contains supplementary material available at 10.1186/s13104-023-06575-w.

## Introduction

The liver acts as an innate immunity-dominant organ and natural killer (NK) cells therefore provide the first line of defense against pathogens, infections or tumors [1, 2]. NK cells are the main lymphocyte population in the human liver accounting for up to 50%, commonly defined by expression of the surface marker CD (cluster of differentiation) 56 and lack of CD3 expression [3, 4]. During liver disease the hepatic NK cell number is modulated, possibly due to increased recruitment of circulating NK cells to the liver [5–7]. A diverse repertoire of NK cell surface receptors allows recognition and rapid response to cell damage and stress [8]. NK cells further coordinate early events of the innate immune response to injury through close interactions with other immune cells, such as T cells, dendritic cells or macrophages [8], and pro-inflammatory cytokine production, such as interferon gamma (IFN-gamma) [3, 8–10], tumor necrosis factor alpha (TNF-alpha) [3, 10], and granulocyte–macrophage colony-stimulating factor [3]. In addition, it has been shown that NK cells produce interleukin-6 (IL-6), TNF-alpha, and IL-1beta after stimulation with lipopolysaccharides (LPS), a common toll-like receptor (TLR) agonist [3]. Furthermore, a variety of surface receptors, such as purinergic receptors and tumor necrosis factor-related apoptosis-inducing ligand, control the cytotoxic activity of NK cells [9, 11] and facilitate NK cell maturation as well as function depend on their cellular localization site [12, 13]. Functionally, NK cells contribute through complex orchestrated pathways, e.g. cytokine or receptor mediated activation, changes of gene expression and interaction with other immune cells (e.g. macrophages), to clearance of damaged hepatocytes during hepatic injury and regeneration [14, 15]. By eliminating susceptible targets through multiple, non-redundant mechanisms, NK cells amplify or reduce the inflammatory response [12]. The close relationship and interaction of NK cells with other immune cells, such as Kupffer cells in the liver sinusoids, is mainly due to cytokine and chemokine secretion [4, 16, 17]. It has been shown that e.g. IL-12 and IL-18, secreted by Kupffer cells, are strong activators of NK cells and necessary for differentiation of NK cells [18–20].

Currently, investigation of NK cell function and hepatic immune cell interaction is limited by the insufficient physiological complexity of available in vitro models. One promising approach is the use of microphysiological systems that allow multicellular organ modelling in a dynamic microenvironment [21–23]. In this study, we expanded a previously established three-dimensional, microfluidically perfused liver-on-a-chip model comprised of human cells (macrophages, endothelial cells, hepatocytes, stellate cells) represents a functional unit of the liver sinusoid and was previously described [24–26]. One study, dealing with taurolidine-induced liver damage, provided comparable results generated from the liver-on-a-chip and an animal model [27]. The overall goal was to integrate human NK cells and show their recruitment and functional response to inflammatory triggers within the liver-on-a-chip. Our study substantiates the use of the developed liver-on-a-chip model for studies of NK cells and paves the way for further immunological in vitro studies.

## Main text

### Isolation of NK cells from human blood and liver specimen

Initially, NK cells were freshly isolated from either human blood or human liver tissue. Specifically, blood-derived NK cell populations were isolated from peripheral blood mononuclear cells (PBMC) using a previously described Percoll separation procedure, followed by PE electromagnetic beads and LS columns sorting, according to manufacturer’s instructions (Miltenyi Biotec, Auburn, CA, USA) [11]. Liver tissue-derived NK cells were isolated from cold-stored, tumor-free liver specimen immediately after surgical resection. Liver specimen were cut into small pieces, passed through a 200 gauge stainless mesh and separated similarly to blood-derived NK cells [28, 29]. The amount and purity of NK cells was assessed by flow cytometry analysis using CD56 (positive selection) and CD3 (negative selection). CD56^+^CD3^−^ NK cell populations from blood or liver tissue were used for further experiments immediately after isolation.

**Fig. 1:**
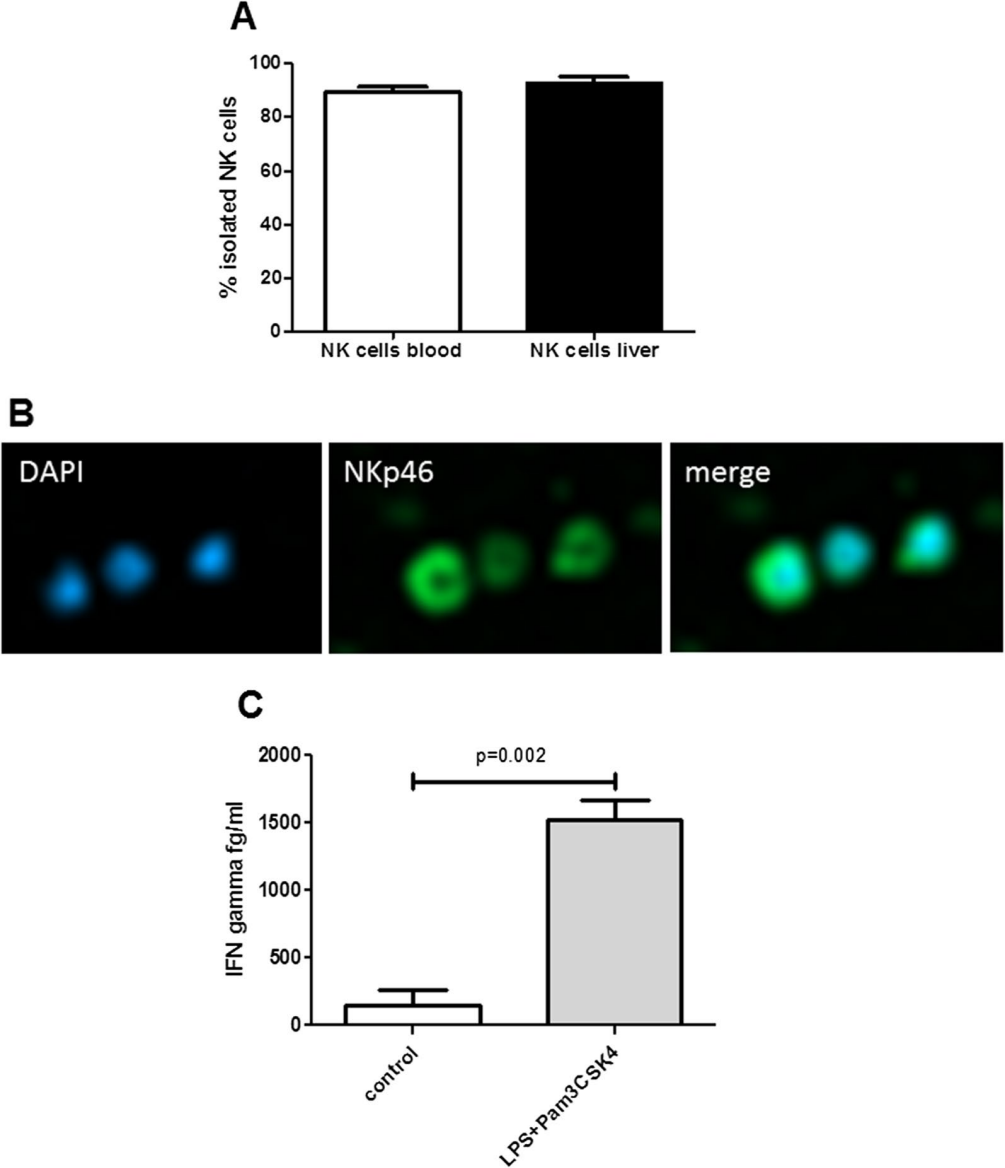
FACS analysis for CD56^+^CD3^−^ NK cells in the NK cell suspension revealed high percentage of isolated NK cells derived from human blood and human liver specimen (**A**). Representative immunofluorescence images of NK cells (**B**) stained with cytotoxicity and maturation marker NKp46 in monoculture of isolated blood NK cells (green = NKp46, blue = DAPI). IFN-gamma secretion assessed by Cytometric Bead Array (CBA) assay (BD Biosciences) of isolated NK cells after 16 h of TLR-receptor agonist stimulation (LPS and Pam3CSK4) was significantly increased compared to controls (**C**). Figures **A**–**C** present data of at least three independent experiments, p-values are assessed by Mann Whitney test

For both isolation procedures, purity of NK cell populations were 89.3 ± 4.9% and 92.7 ± 4.6%, respectively (Fig. 1A), comparable to previous reports [29].

NKp46, a marker of NK cell cytotoxicity and maturation [30, 31], was expressed on isolated NK cells, which was verified by immunofluorescence staining (Fig. 1B and Additional file 1: Fig. S1). To activate NK cells in vitro, these cells can be stimulated with a macrophage-derived cytokine mixture (e.g. IL-12/18) or TLR agonists [3, 9]. To activate isolated NK cells, they were challenged with TLR-agonists (LPS and Pam3CSK4), which were previously used to induce inflammation in the liver-on-a-chip model. As functional readout IFN-gamma was used, as one of the most important cytokines secreted by NK cells. IFN-gamma was measured in the supernatant of cell cultures using the cytometric bead array (CBA; BD Biosciences) according to the manufacturer’s protocol. After stimulation of isolated NK cells with LPS (100 ng/ml) und Pam3CSK4 (250 ng/ml) for 16 h, IFN-gamma secretion was significantly increased in comparison to controls reflecting the activation and functionality of isolated NK cells (Fig. 1C).

### Integration of NK cells in the liver-on-a-chip model

Human organ on-a-chip models enable the investigation of physiological and pathophysiological processes in vitro in a standardized, multicellular microenvironment. These complex in vitro models offer advantages compared to common in vitro and animal models through scalability and replication of organ-specific microenvironments ex vivo. Furthermore, biochip-embedded models obtain an optimal supply with oxygen and nutrients due to microfluidic media perfusion of the chip [26]. This allows more physiological, multicellular tissue culture improving cellular communication and functional polarization [21]. Conventional cell cultures may overcome some species-dependent barriers, but are limited to reflect physiological conditions in respect to e.g. complex cellular cross-communications [21]. Furthermore, improved culture conditions in organ-on-a-chip models also allow integration of circulating immune cells. Although conventional cell cultures will still remain the mainstay of cellular in vitro analysis, the organ-a-chip technology will help to investigate complex cellular interactions in more physiological conditions [21].

The underlying biochip-based model represents a three-dimensional functional unit of the liver with a permeable membrane serving as a cell culture surface and artificial barrier, allowing microfluidic perfusion and physiological arrangement of cellular structures in vitro. This liver-on-a-chip model used for our analysis was previously established and characterized, showing functional improvements compared to conventional in vitro culture [24, 27]. The so called multi-organ-tissue-flow (MOTiF) biochip, which is manufactured using a cyclic olefin copolymer (COC), was purchased from ChipShop GmbH (Jena, Germany) as previously described [25, 26]. Briefly, the biochip design with two channels and one 12 µm thick PET membrane (pore diameter 8 µm with a density of 2 × 10^5^ pores/cm^2^) allows precise control of flow conditions maintained through perfusion of media through silicon tubes connected to a peristaltic pump. To reduce bubble formation in the biochip, ramping structures were introduced and chips were hydrophilized by oxygen plasma treatment. Top and bottom side of the chip and channel structures were sealed by a 140 µm thick COC film. Prior to seeding of all cells, biochips were equilibrated overnight by media perfusion of the upper (vascular/endothelial layer) and lower (epithelial layer) channel of the chip for 24 h. The human liver-on-a-chip was assembled by seeding of endothelial cells (human umbilical vein endothelial cells) as well as resident primary macrophages (vascular layer, upper channel) and hepatocytes (HepaRG) as well as stellate cells (LX2; epithelial layer, lower channel) as previously described [27]. In human livers, NK cells are located next to macrophages, lymphocytes and myeloid cells in the endothelial layer, leading to close interactions of these immune cells [4, 32]. To mimic the physiological state, isolated blood derived NK cells were added to the vascular layer in a ratio of one to two, NK cells to Kupffer cells, respectively. The upper, vascular chamber was continuously perfused with media mimicking the blood flow, whereas the bottom, epithelial chamber was kept static with daily media exchange.

### Perfusion and stimulation of liver-on-a-chip model comprising NK cells

**Fig. 2:**
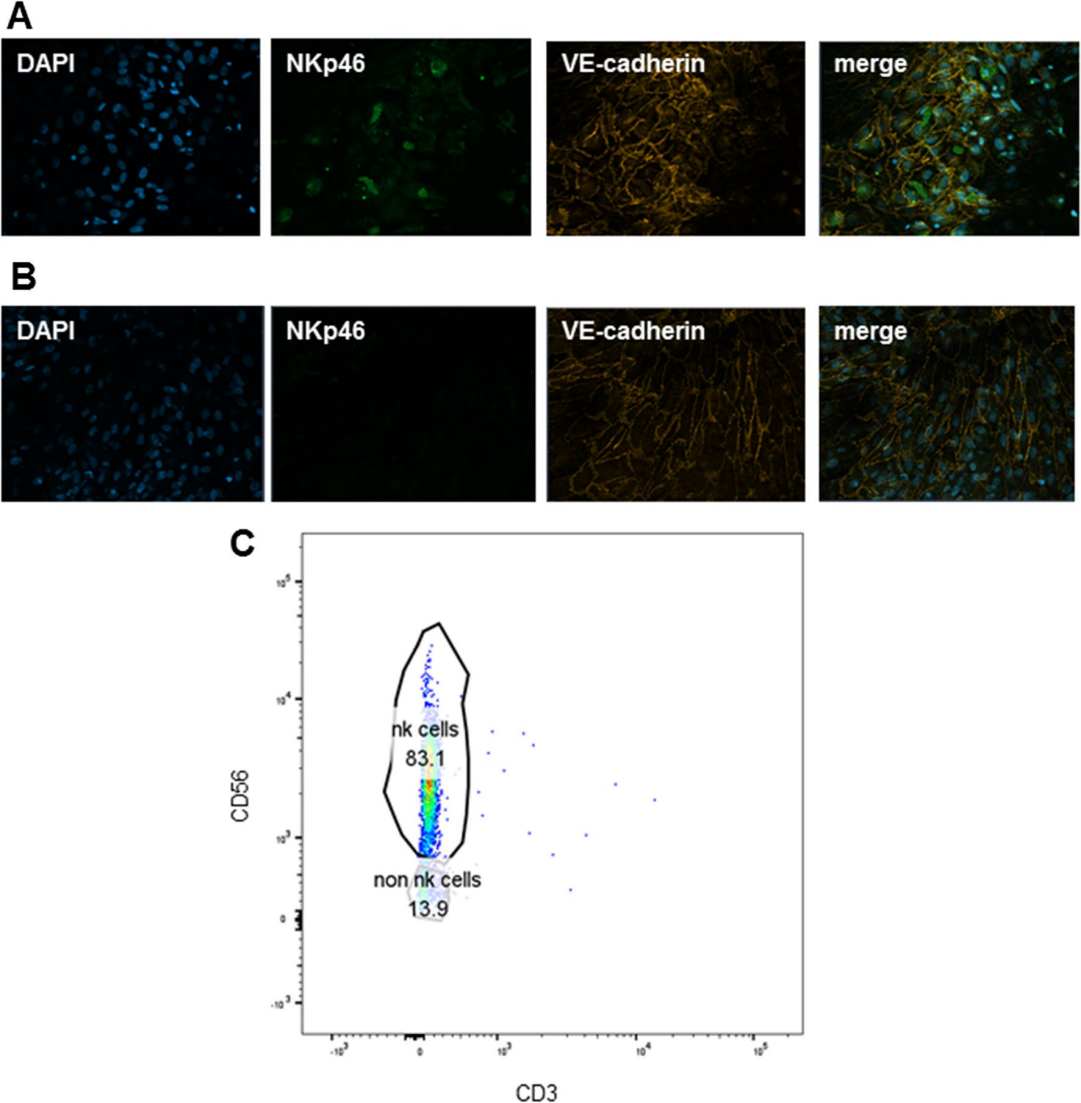
Representative immunofluorescence image (**A**) of the endothelial layer including NK cells and without NK cells (**B**) stained with cytotoxicity and maturation marker NKp46 (green = NKp46, blue = DAPI, orange = VE-cadherin). Detection of CD56^+^/CD3^−^ NK cells by FACS isolated from the endothelial layer of the liver-on-a-chip model after 48 h of perfusion (**C**)

**Fig. 3:**
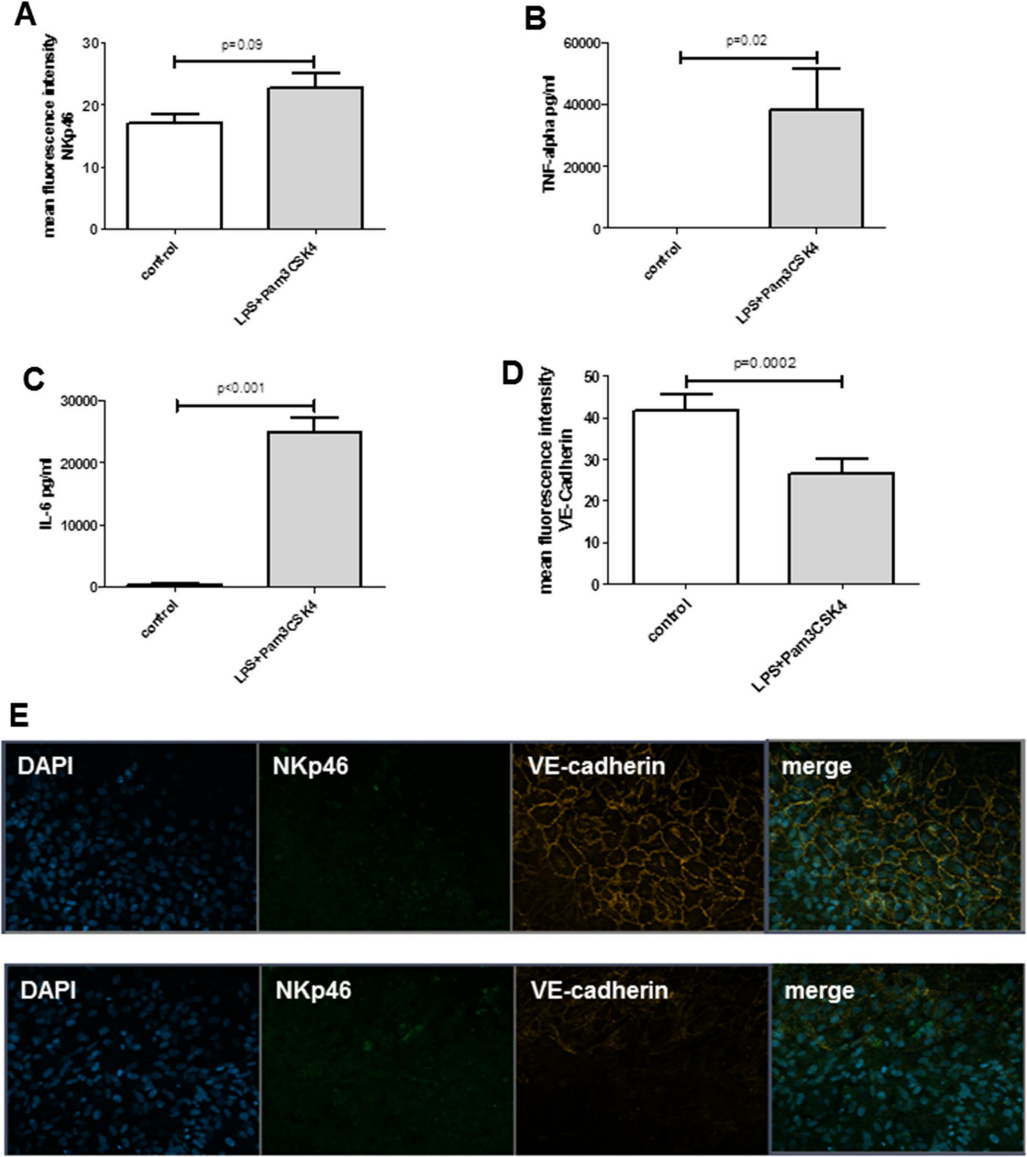
Stimulation with TLR-receptor agonists (LPS and Pam3CSK4) revealed a trend to higher expression of the maturation and cytotoxicity marker NKp46 on the endothelial layer of the liver-on-a-chip model (**A**). Increased TNF-alpha (**B**) and IL-6 (**C**) secretion of liver-on-a-chip including NK cells after 24 h perfusion with TLR-receptor agonists. Liver injury, reflected by endothelial cell layer disruption, assessed by immunofluorescence of VE-cadherin, was significantly increased after TLR-receptor agonist stimulation than in controls (**D**). Representative immunofluorescence image (**E**) showing disruption of the endothelial layer after application of the TLR receptor agonists. Figures **A**–**D** present data of at least three independent experiments, p-values are assessed by Mann Whitney test

The main goal of this project was, to integrate functional NK cells into the existing liver-on-a-chip model. Therefore, freshly isolated blood NK cells were seeded as described into the liver-on-a-chip model. After 48 h of perfusion, the presence of NK cells in the endothelial cell layer was verified by immunofluorescence staining, analysis of the maturation and cytotoxicity marker NKp46 (Fig. 2A, B), and flow cytometry analysis. Hence, cells of the vascular layer were detached from the membrane and the NK cell population was analyzed by flow cytometry using CD3 (Horizon VE450), CD16 (PE), and CD56 (PE-Cy7). The NK cell population was defined by expression of NK cell surface markers CD56 and absence of CD3 (Fig. 2C). To prove that NK cells were still responsive to inflammatory triggers, the liver-on-a-chip model was cultured for a maximum 48 h with and without TLR-agonists (LPS/Pam3CSK4) stimulation. Under physiological conditions, the liver is constantly exposed to antigens, pathogens, and tumor cells, entering the liver via the blood flow to the liver sinusoids. The immune and endothelial cells are first exposed to these agents, and thus build the first line of defense [32, 33]. Comparable to the NK cells stimulation in monocultures, the hepatic injury was induced by TLR stimulation through continuous perfusion of the upper chamber consisting of endothelial cells, NK cells and macrophages. Interestingly, a trend of increased expression of NKp46 after TLR stimulation compared to control was observed (p = 0.09, Fig. 3A), indicating an increased number and associated cytotoxic activity of NK cells in the model. Furthermore, the pro-inflammatory cytokines IL-6 (p = 0.005) and TNF-alpha (p = 0.03) were significantly increased after TLR stimulation compared to controls (Fig. 3B, C), potentially due to secretion by NK cells and macrophages. Nevertheless, IFN-gamma secretion was under the lower detection limit of the assay in the liver-on-a-chip-model after 16 and 24 h of TLR agonist stimulation, respectively. These low IFN-gamma levels may be a result of NK cell differentiation or protective feedback mechanisms from other cell types in the liver-on-a-chip model leading to different NK cell function and cytokine secretion [1, 34]. TLR-stimulation of the liver-on-a-chip model further led to a disruption of the endothelial barrier assessed by the significantly reduced expression of VE-cadherin compared to the control condition (p = 0.05, Fig. 3D, E).

In conclusion, our data highlights the first successful approach of integrated viable und functional NK cells isolated from human blood in a liver-on-a-chip model. NK cells and other immune cells, e.g. macrophages, have close interactions during inflammatory and immunological processes in the liver. The aim of this investigation was to establish an in vitro model including NK cells representing a functional hepatic unit as close as possible to the in vivo situation. Our study highlights the potential of organ-on-a-chip models to specifically analyze the contribution of isolated immune cells under physiological conditions, which is currently only possible using animal models or human studies. Further functional analysis and cell interactions of NK cells in the liver-on-a-chip model need to be conducted to provide a more comprehensive analysis of pathophysiological mechanisms. Future applications could be to investigate NK cells driven immunological processes during hepatic inflammation (sterile or bacterial induced) with the advantage of a controlled and standardized in vitro model comprising of human cells.

## Limitations

NK cells were successfully isolated from human blood and human livers, but so far only human blood NK cells and not liver NK cells were integrated into the existing liver-on-a-chip model. Previous reports showed functional differences between liver-resident NK cell populations and recruited NK cells from the blood invading the liver [7, 35]. Follow-up studies are necessary to determine the differences between different NK-cell populations in our liver-on-a-chip model. So far, we could show, that the transfer of freshly isolated blood-derived NK-cells into the liver-on-a-chip-model was possible, but further functional investigations and differentiation or maturation processes of NK cells [36] were not investigated. In addition, studies without resident macrophages and only NK cells should elucidate the specific contribution of NK cells in the liver during inflammation. These issues are possible cornerstones of future studies using this presented model.

### Supplementary Information

**Additional file 1: Figure S1.** Representation immunofluorescence images of NK cells in low magnification stained with cytotoxicity and maturation marker NKp46 in monoculture of isolation blood NK cells (green=NKp46, blue=DAPI, nuclei).

## Data Availability

The datasets used and/or analyzed during the current study are available from the corresponding author on reasonable request.

## Abbreviations

CD: Cluster of differentiation
COC: Cyclic olefin copolymers
FACS: Fluorescence-activated cell sorting
IL: Interleukin
LPS: Lipopolysaccharides
MOTiF: Multi-organ-tissue-flow
NK: Natural killer
PBMCs: Peripheral blood mononuclear cells
TNF: Tumor necrosis factor
TLR: Toll-like receptor
