# Supplementary figures and images for: Functional integration of natural killer cells in a microfluidically perfused liver on-a-chip model
Source: BMC Res Notes. 2023 Oct 21;16:285. doi: 10.1186/s13104-023-06575-w (PMC10590007; doi:10.1186/s13104-023-06575-w)

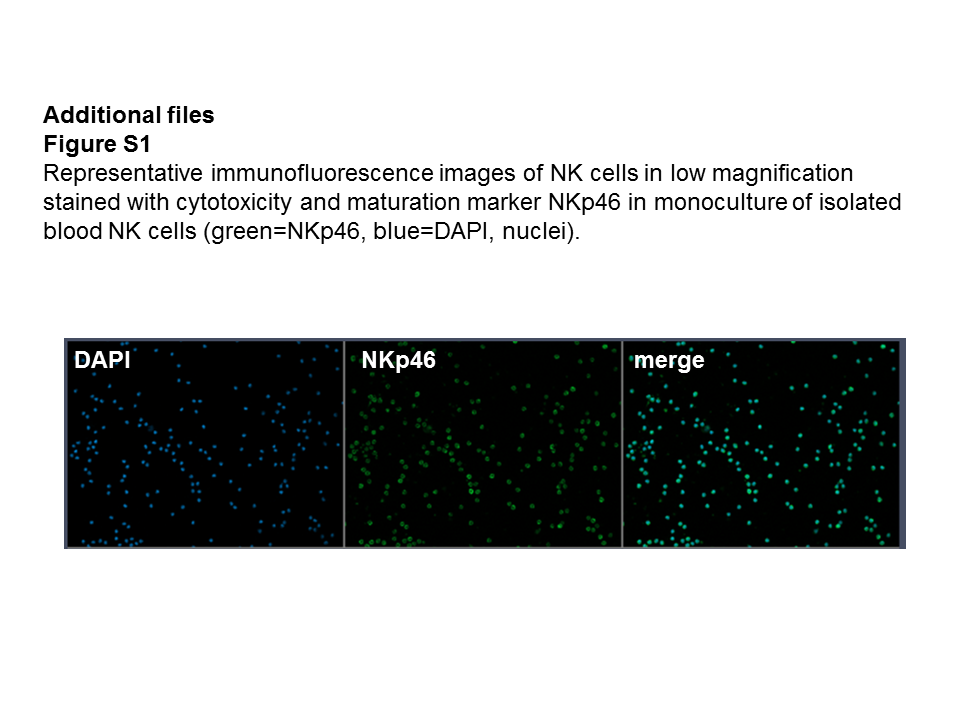

Supplement: Supplementary file 1 — Additional file 1: Figure S1. Representation immunofluorescence images of NK cells in low magnification stained with cytotoxicity and maturation marker NKp46 in monoculture of isolation blood NK cells (green=NKp46, blue=DAPI, nuclei). [file 13104_2023_6575_MOESM1_ESM.tif]
